# Characterization of *Salmonella enterica* serovars recovered from meat products legally and illegally imported into the EU reveals the presence of multiresistant and AmpC-producing isolates

**DOI:** 10.1186/s13099-018-0268-3

**Published:** 2018-09-22

**Authors:** Anja Müller, Wiebke Jansen, Nils Th. Grabowski, Corinna Kehrenberg

**Affiliations:** 1 Institute of Food Quality and Food Safety, University of Veterinary Medicine Hannover Foundation, Bischofsholer Damm 15, 30173 Hannover, Germany; 2 Integrated Veterinary Research Unit, University of Namur, Rue de Bruxelles 61, 5000 Namur, Belgium

**Keywords:** AmpC-β-lactamase, Antimicrobial resistance, Legal import, Illegal import, Meat products, *Salmonella enterica*

## Abstract

**Background:**

Food products of animal origin brought into the EU from third countries, both legally and illegally, can harbor foodborne pathogens such as *Salmonella enterica*. In this study, we examined five *S. enterica* isolates recovered either from legally imported chicken meat (n = 3) or from meat products confiscated from air travel passengers arriving in Germany (n = 2). The isolates were serotyped and further characterized by antimicrobial susceptibility testing, PCR-detection and sequencing of genes associated with antimicrobial resistances, and macrorestriction analysis. Transferability of resistance to third-generation cephalosporins was assessed by conjugation experiments and the plasmids tested for their incompatibility groups.

**Results:**

The three isolates from legal imports were identified as *S*. Heidelberg or as non-flagellated. All three isolates were identified as AmpC producers carrying *bla*_CMY-2_ and as non-susceptible to ciprofloxacin. They were additionally resistant to tetracycline and sulfamethoxazole. The *bla*_CMY-2_-carrying plasmids were transferable by conjugation and belonged to incompatibility groups IncI1 or IncA/C. The two isolates from illegally imported meat belonged to the serovars Infantis or Weltevreden. The former was phenotypically resistant to five classes of antimicrobial agents while the *S*. Weltevreden isolate was fully susceptible to all agents tested.

**Conclusion:**

The results of this study demonstrate that meat products imported from third countries, both legally and illegally, can harbor multiresistant *Salmonella enterica*. Consequently, these imports could constitute a source for the dissemination of antimicrobial resistant isolates, including those resistant to third-generation cephalosporins and fluoroquinolones.

## Background

*Salmonella* (*S*.) *enterica* is one of the most common bacterial pathogens causing foodborne infections and, thus, constitutes a major healthcare concern worldwide. For 2016, the European Food and Safety Authority (EFSA) reported over 94,000 confirmed cases of human salmonellosis in the European Union (EU) and *S. enterica* was identified as the bacterial agent responsible for the most foodborne outbreaks [1]. Poultry meat constitutes an important source of *Salmonella enterica* and infections in humans are often linked to the consumption of improperly cooked poultry meat or a cross-contamination of other foodstuffs [1]. While most non-typhoidal *Salmonella* spp. infections lead to self-limiting gastrointestinal symptoms, invasive infections can occur and may be life-threatening, particularly in immunocompromised patients [2, 3]. However, the likelihood of severe infections differs between serovars. *S.* *enterica* serovar Typhimurium, which is among the most frequently encountered serovars in the EU, has a moderate tendency to cause invasive disease—*S*. Typhimurium ST313 being a notable exception, frequently associated with invasive disease in Africa [4]. Others, such as *S.* Heidelberg and narrow host-range serovars like *S.* Dublin and *S*. Choleraesuis, are considerably more likely to cause severe infections requiring hospitalization [3, 5]. Previous reports of salmonellosis outbreaks involving multiple countries have raised concerns about the role of international food and animal trade in the spread of *Salmonella* serovars, especially considering the comparatively high rates of *Salmonella* detection in some parts of the world [6]. Furthermore, imported meat products have been implicated as a possible source for *S. enterica* isolates resistant to clinically relevant antimicrobials [7, 8].

In addition to consignments of meat officially imported into the EU, considerable amounts of meat products are brought into the EU illegally, circumventing any controls [9, 10]. To date, very little data are available about serovars present in such products and their characteristics [11, 12].

In this study, *S. enterica* isolates recovered from meat and meat products imported into the EU, both legally and illegally, were examined. The purpose was to identify the serovars entering the EU via these routes and to further characterize the isolates regarding their antimicrobial resistance phenotypes and genotypes.

## Methods

### Bacterial isolates

A total of five *Salmonella enterica* isolates were examined in this study. They were collected in the years 2014 and 2015, in the course of a previous study (Project “ZooGloW”) investigating meat and meat products imported into the European Union both legally and illegally. The procedures for the isolation of *Salmonella* spp. performed in that study and the sampling of legal poultry meat and pork imports have been described previously [13]. In brief, 516 poultry meat and 136 pork samples, cleared via the border inspection post Hamburg Harbour in 2014 and 2015, were subjected to microbiological analyses. The samples were taken aseptically by the competent authorities, stored in individually sealed bags and were subsequently frozen on-site. They were kept at − 18 °C during storage and transport to the laboratory. Detection of *Salmonella* spp. was performed according to DIN EN ISO 6579:2002 with an initial enrichment of a 10 g sample. *Salmonella* spp. isolates were identified in six of these samples from legally imported meat [13].

Following the same procedures, a total of 344 samples of illegally imported meat products, seized from air travel passengers by the competent authorities at Berlin Schönefeld Aiport (SXF) and Frankfurt International Airport (FRA), were examined (unpublished data). These samples included raw and processed products made of pork (n = 203), poultry (n = 127), beef (n = 8), lamb (n = 4), chamois buck (n = 1), rabbit (n = 1) and one dried meat sample of an unknown species. The presence of *Salmonella enterica* could be confirmed in five samples of raw poultry meat (n = 4) or rabbit meat (n = 1).

**Fig. 1:**
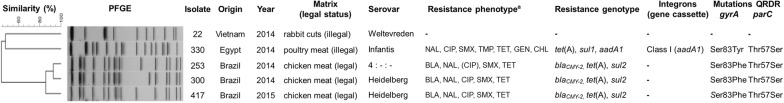
Typing results, resistance phenotypes and genotypes of the five *Salmonella* isolates. ^a^Only antimicrobial agents for which CLSI-approved MIC breakpoints are available, are included. *BLA* β-lactams, *CHL* chloramphenicol, *CIP* ciprofloxacin, *GEN* gentamicin, *NAL* nalidixic acid, *SMX* sulfamethoxazole, *TET* tetracycline, *TMP* trimethoprim, brackets indicate intermediate resistance

However, three isolates each from legal imports and illegally imported meat products could not be successfully recultured after cryopreservation. Consequently, a total of five of the previously collected *Salmonella* isolates were available for further analyses in the course of the current study. These included three isolates from legal imports, which were recovered from three separate batches of chicken meat, which traced back to producers operating under different registration numbers each. The remaining two isolates were recovered from illegally imported raw meat, comprising one sample of rabbit cuts (belly, ribs and shoulder) and one sample of marinated poultry meat. An overview of the origin of the samples is given in Fig. 1.

### Serotyping and macrorestriction analysis

The isolates were sent to the National Reference Centre for Salmonellae and other Bacterial Enteric Pathogens (Robert Koch Institute, Wernigerode, Germany) for serotyping according to the White-Kauffmann-Le Minor scheme and phage typing of *S*. Infantis. Phage typing was performed according to the typing system of Miller et al. [14].

The genetic relatedness of the isolates was determined by macrorestriction analysis with *Xba*I digestion followed by pulsed-field gel electrophoresis (PFGE) according to the PulseNet protocol for *Escherichia coli* O157:H7, *Salmonella*, and *Shigella* [15]. The Bionumerics software version 7.6 (Applied Maths, Sint-Martens-Latem, Belgium) was used for band pattern analysis, applying the Dice coefficient with 0.5% optimization and 1% position tolerance.

### Antimicrobial susceptibility testing and detection of resistance genes

Minimum inhibitory concentration values (MICs) were determined according to Clinical and Laboratory Standards Institute (CLSI) documents for broth microdilution and broth macrodilution susceptibility testing [16, 17]. Microtiter plates Micronaut-S for large animals (“Großtiere”), containing 19 antimicrobial agents or combinations, and Micronaut-S β-lactamases (Merlin Diagnostika, Bornheim-Hersel, Germany) were used. *Escherichia coli* ATCC25922 and ESBL-producing *K. pneumoniae* ATCC700603 served as quality control strains.

PCR assays targeting genes associated with antimicrobial resistances were carried out with previously described primers and protocols. The PCR assays included β-lactamase encoding genes *bla*_TEM_, *bla*_CTX-M_, *bla*_CMY-2_, *bla*_OXA-1_-like, *bla*_OXA-2_, and *bla*_SHV_ [18, 19]; aminoglycoside resistance determinants *ant*-*(*2″)-I, *aac*(3)-II, *aac*(3)-IV [20]; tetracycline resistance genes *tet*(A), *tet*(B), *tet*(C), *tet*(D), *tet*(E), *tet*(G), *tet*(H), *tet*(M), *tet*(O) [18]; quinolone resistance-associated genes *qnrA*, *qnrB*, *qnrC*, *qnrD*, *qnrS*, *aac*(6′)-*Ib*-*cr*, *qepA* [21, 22]; phenicol resistance genes *cmlA, catA1, catA2, catA3, catB2, catB3*, *cfr*, *fexA*, *floR*, [18, 23]; as well as *sul1*, *sul2*, *sul3* and *dfrA1*, *dfrA5/14*, *dfrA7/17*, *dfrB1/2* [18], conferring resistance to sulfonamides and trimethoprim, respectively. Additionally, the isolates were tested for the presence of integron classes I, II and III [24, 25]. The quinolone-resistance determining regions of *gyrA*, *gyrB*, *parC*, *parE* were amplified with previously described primers [26]. Sequencing of amplicons obtained for *bla*_CMY_, *gyrA*, *gyrB*, *parC*, *parE* and the variable regions of integron class I was carried out by Eurofins Genomics (Ebersberg, Germany). Sequences were analyzed using the DNAMAN software version 8 (Lynnon Biosoft, San Ramon, CA, USA) and the BLASTN algorithm [27]. Incompatibility groups of *bla*_CMY-2_-carrying plasmids were determined by PCR as described previously [28].

### Conjugation experiments

**Fig. 2:**
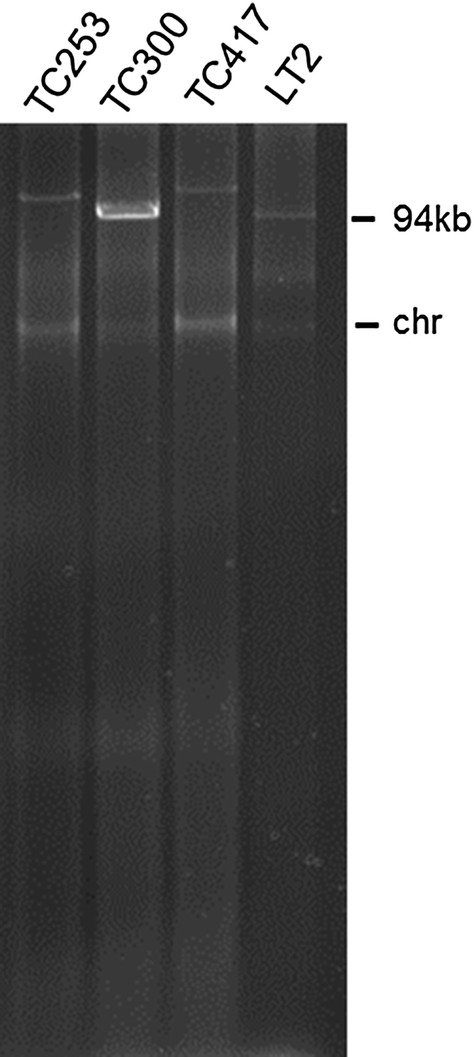
Plasmid profiles of HK225 transconjugants, run in a 1% agarose gel and stained with ethidium bromide. AmpC-producing *Salmonella enterica* isolates were used as donor strains. The 94 kb plasmid of *S*. *Typhimurium* strain LT2 served as size marker. *Chr* residues of chromosomal DNA

Isolates with resistance to third-generation cephalosporins were subjected to conjugation experiments with plasmid-free, rifampicin- and streptomycin-resistant *Escherichia coli* HK225 as recipient strain [29]. Donor and recipient strain were grown overnight in 10 ml LB medium containing ampicillin (50 µg/ml) or rifampicin (100 µg/ml), respectively. Following centrifugation and resuspension of the pellet in LB without antibiotics, the cultures were mixed in a 1:5 ratio (donor:recipient) based on their optical density. The mixture was centrifuged, the pellet transferred onto LB agar and incubated overnight at 35 °C. The following day, the conjugation mixture was recovered from the agar plate and suspended in 1 ml LB broth. Serial dilutions were plated on LB agar, LB agar supplemented with rifampicin (100 µg/ml), LB agar supplemented with ampicillin (50 µg/ml), and LB agar supplemented with rifampicin and ampicillin (100 µg/ml and 50 µg/ml, respectively). Transconjugants were taken from the double selective plates and confirmed as *E. coli* by PCR targeting the *gadA* gene [30]. Plasmids were extracted using a modification of the alkaline lysis method as described previously [31]. Plasmid sizes were estimated by agarose gel electrophoresis and visual comparison to the 94 kilobase pairs (kb) plasmid of *S*. Typhimurium stain LT2 (Fig. 2) [32]. One representative transconjugant per isolate, carrying a single plasmid, was chosen for further analyses.

## Results

### Serotypes and antimicrobial resistances

Among the isolates recovered from legally imported samples, two were identified as *S*. Heidelberg whereas the third was a non-flagellated, d-tartrate-positive isolate of O group B (O:4). However, all three isolates showed a high similarity of PFGE band patterns of up to > 90% (Fig. 1). The two isolates from illegally imported meat showed less than 50% similarity with the three isolates from legal imports and each other. The isolate from illegally imported meat from Vietnam was identified as *S*. Weltevreden. The second isolate, recovered from marinated poultry meat illegally introduced from Egypt, belonged to the serovar Infantis. This isolate showed a lysis pattern that did not conform to any recognized *S*. Infantis phage type and was designated “reacts but does not conform” (RDNC).

All isolates recovered from legal imports and one isolate from illegally imported meat were resistant to three or more classes of antimicrobial agents and were classified as multiresistant [33]. The respective resistance phenotypes and genotypes are shown in Table 1. The three isolates from legal imports were AmpC-producers and carried *bla*_CMY-2_. They were further resistant to nalidixic acid, sulfamethoxazole, and tetracycline and either resistant (n = 2) or intermediate resistant (n = 1) to ciprofloxacin (MICs of 1 µg/ml or 0.5 µg/ml ciprofloxacin, respectively), according to CLSI breakpoints. Decreased quinolone susceptibility was attributed to mutations resulting in a Ser83Phe substitution in the deduced amino acid sequence of the GyrA protein and a Thr57Ser substitution in ParC (Fig. 1). No isolate carried the genes *qnrA*, *qnrB*, *qnrC*, *qnrD*, *qnrS*, *aac*(6′)-*Ib*-*cr,* or *qepA*, associated with plasmid-mediated quinolone resistance.

**Table 1:** Characteristics and comparison of AmpC-producing *Salmonella* donor strains and *Escherichia coli* transconjugants

Isolate	Resistance genes	Inc. group of transferred Plasmid	Resistance phenotype	MIC values (µg/ml)							
				AMP	AMC	CTX	CAZ	NAL	CIP	SMX	TET
253	*bla*_CMY-2_, *tet*(A), *sul2*	IncA/C	BLA, NAL, (CIP), SMX, TET	> 64	> 32/16	> 4	> 8	> 128	0.5	> 1024	> 64
TC253 (HK225 transconjugant)	*bla*_CMY-2_, *tet*(A), *sul2*		BLA, SMX, TET	> 64	> 32/16	> 4	> 8	8	0.06	>1024	32
300	*bla*_CMY-2_, *tet*(A), *sul2*	IncI1	BLA, NAL, CIP, SMX, TET	> 64	> 32/16	> 4	> 8	> 128	1	> 1024	> 64
TC300 (HK225 transconjugant)	*bla*_CMY-2_		BLA	> 64	> 32/16	> 4	> 8	8	0.06	32	≤ 2
417	*bla*_CMY-2_, *tet*(A), *sul2*	IncA/C	BLA, NAL, CIP, SMX, TET	> 64	> 32/16	> 4	> 8	> 128	1	> 1024	> 64
TC417 (HK225 transconjugant	*bla*_CMY-2_, *tet*(A), *sul2*		BLA, SMX, TET	> 64	> 32/16	> 4	> 8	8	0.06	>1024	32
*E. coli* HK225	–	–	–	2	≤ 2/1	≤ 0.25	≤ 0.5	8	0.06	32	≤ 2

*AMC* amoxicillin/clavulanate (2:1), *AMP* ampicillin, *BLA* β-lactams, *CAZ* ceftazidime, *CIP* ciprofloxacin, *CTX* cefotaxime, *NAL* nalidixic acid, *SMX* sulfamethoxazole, *TET* tetracycline

The *S*. Infantis isolate from illegally imported meat also showed multiple phenotypic resistances. It was resistant to nalidixic acid, ciprofloxacin, tetracycline, gentamicin, sulfamethoxazole, trimethoprim, chloramphenicol and additionally showed elevated MIC values for florfenicol (MIC = 32 µg/ml). Furthermore, this isolate harbored a class I integron containing an *aadA1* gene cassette showing 100% nucleotide sequence identity to previously published sequences (e.g. GenBank KF921523). The isolate did not produce positive results with any of the primer pairs targeting trimethoprim, gentamicin and phenicol resistance genes included in this study, however. In contrast to this, the *S*. Weltevreden isolate was susceptible to all antimicrobial agents tested.

### Localization of cephalosporin resistance genes on transferable plasmids

Mating experiments demonstrated inter-genus transferability of the plasmid bearing *bla*_CMY-2_ for all three AmpC-producing isolates. The *bla*_CMY-2_ genes of isolates 253 and 417 were located on an IncA/C plasmid with an estimated size of over 100 kb, which further contained *sul2* and *tet*(A), rendering *E. coli* HK225 transconjugants phenotypically resistant to tetracycline and sulfonamides in addition to β-lactams. The efficiency of conjugation of these plasmids was rather low, however, with 1.23 × 10^−7^ and 3.8 × 10^−8^ transconjugants per donor for isolates 253 and 417, respectively. The plasmid of the third isolate belonged to incompatibility group IncI1 and was transferred at a markedly higher frequency (5 × 10^−3^). This plasmid was slightly smaller with an estimated size of over 95 kb, based on visual comparison to the 94 kb plasmid of *S*. Typhimurium LT2. No co-transfer of other resistance determinants was observed for this plasmid.

## Discussion

In recent years, the number of air travel passengers has increased continuously and is expected to rise to an estimated 7.8 billion travelers in 2036 [34]. Consequently, the total amount of meat and meat products illegally transported in passenger luggage can be expected to rise accordingly. To date, little data are available about the characteristics of salmonellae recovered from such food items. In the present study, the two isolates from illegally imported meat belonged to the serovars Infantis or Weltevreden. Both isolates were recovered from raw meat products, in one case seasoned with a marinade of unknown composition, transported in air passenger luggage. In contrast to this, a study conducted in Spain reported the following seven different serovars among eleven isolates from food of animal origin confiscated at a Spanish airport: monophasic serovar 4, 12: d: − , Rauform, Anatum, Oranienburg, Enteritidis, Newport and Typhimurium [11]. Notably, these were mostly of South American origin and included isolates detected in cheese samples. Counting only meat and meat products, 6 of the 122 samples examined in that study were positive for *S. enterica* [11]. In another study, a total of four isolates were detected on illegally introduced sausages of Russian origin out of 367 samples of illegally imported meat and meat products (1.1%) [12]. Two isolates each belonged to the serovars *S*. Infantis and *S*. Enteritidis. In their study, as well as in the course of the sampling preceding the current study, samples were kept frozen before analyses [12, 13]. Previous research demonstrates that freezing does not fully eliminate *S. enterica,* but provides differing results on survival rates during frozen storage ranging from no significant changes in bacterial counts to minimal survival [35, 36]. Consequently, structural injury to the bacterial cells during freezing and thawing of the samples prior to analyses may have contributed in part to the low detection of *Salmonella* spp.. One study reported a relatively constant population of *Salmonella* spp. isolates in artificially inoculated breaded chicken strips and nuggets during 16 weeks of storage at − 20 °C. However, enumeration on selective agar resulted in a statistically significant reduced recovery of salmonellae compared to non-selective media, indicating structural injury to the cells [35]. In contrast to this, the authors of a study conducted in Mexico observed a considerable decline of the S*almonella* population present on naturally contaminated pork during storage at − 15 °C [36]. In a recent study, a lower detection rate of salmonellae in frozen chicken carcasses was determined compared to chilled and non-chilled samples, though the difference was not statistically significant [37]. Due to different storage conditions and meat types used in individual studies, the comparability of the results is limited. Additionally, many further factors were suggested to influence the survival of *Salmonella* spp. during frozen storage, such as pH and fat content of the meat, which were not evaluated for the samples examined prior to the current study [35].

*S*. Weltevreden is a common serovar in Southeast Asia and the isolate in the present study was recovered from illegally imported rabbit meat from Vietnam [38, 39]. The isolate did not show resistances to any of the antimicrobial agents included in the test panel. This is in accordance with previous studies that found a low prevalence of antimicrobial resistances for this serovar [40, 41]. Of the 11 *S. enterica* isolates from illegally imported food products of animal origin examined in the study by Rodríguez-Lázaro and collegues, most isolates did not show phenotypic resistances to any of the antimicrobial agents tested, either. The remaining isolates were each resistant to a single class of antimicrobials [11].

In contrast to this, the *S*. Infantis in the present study was resistant to substances belonging to five different classes of antimicrobials. In recent years, resistances to multiple antimicrobial agents have been observed comparatively frequently in *S*. Infantis. Together with *S*. Kentucky, this serovar currently accounts for a significant proportion of multiresistant non-typhoidal *S*. *enterica* isolates in the EU [42].

The isolates from legally imported chicken meat in the current study included one non-flagellated isolate. However, this isolate appeared to be closely related to the two *S*. Heidelberg isolates recovered from legal imports, based on the high similarity in PFGE band patterns and the shared O-group. Flagella play an important role as virulence factors and enhance adhesion to and invasion of host cells. However, this mostly affects the early stages of infection and non-flagellated strains do not appear notably less virulent if an infection reaches the systemic phase [43, 44].

*S*. Heidelberg is not commonly associated with human cases of salmonellosis in the EU and did not rank among the 20 most frequent serovars in an EFSA report covering the years 2014–2016 [1]. Data on national cases of human salmonellosis, released by the Robert Koch Institute, also indicate a low prevalence of *S*. Heidelberg infections in Germany [45]. According to these data, the four serovars accounting for the vast majority of cases in 2016 were *S*. Enteritidis and *S*. Typhimurium, followed by *S*. Infantis and *S*. Derby. Particularly in North America, however, *S*. Heidelberg is often the cause of human salmonellosis and was reported to be the sixth most frequent serovar in the US in 2015 [46, 47].

Notably, all isolates from legal imports in this study were recovered from fresh poultry meat. Regarding *Salmonella*, commission regulation (EC) 2073/2005, amended by commission regulation (EU) 1086/2011, only specifies the absence of *S*. Enteritidis and *S*. Typhimurium (including its monophasic variant) as food safety criteria for fresh poultry meat. Other serovars are not covered, even though they might be more common in other countries such as Brazil – the largest exporter of chicken meat supplying the EU [8, 48–50].

All three isolates from legally imported poultry meat in this study were AmpC producers carrying *bla*_CMY-2_ and they were additionally resistant to ciprofloxacin. Like third-generation cephalosporins, fluoroquinolones are important choices for the treatment of severe cases of salmonellosis [51]. Consequently, co-resistance to both is of particular concern. The overall prevalence of ESBL- and AmpC-β-lactamases among *Salmonella enterica* isolates is still low in the EU, however, the AmpC phenotype in particular has been comparatively often seen in *S*. Heidelberg isolates [52, 53]. *S*. Heidelberg carrying *bla*_CMY-2_ have previously been detected on imported poultry meat of Brazilian origin and such imports were suggested to be involved in the increase of third-generation cephalosporin-resistant *S*. Heidelberg in the Netherlands [7, 8]. The *bla*_CMY-2_ genes of the isolates in the present study were located on large conjugative plasmids belonging either to IncA/C or IncI1. Plasmids of these incompatibility groups have previously been associated with AmpC-producing *S.* Heidelberg from various sources and likely play an important role in the spread of resistance to third-generation cephalosporins in *S*. Heidelberg and other *Salmonella* serovars [7, 8, 47, 54].

## Conclusion

In conclusion, this study reports the presence of multiresistant *Salmonella enterica* isolates in meat products imported into Germany either legally or illegally. In particular, the co-resistance to third generation cephalosporins and fluoroquinolones in all three isolates from legally imported meat is worrying. Despite the limited number of isolates examined, the results of this study demonstrate how meat and meat products entering the EU could constitute a route for the dissemination of such isolates and provides further evidence that serovars other than Enteritidis and Typhimurium on fresh poultry meat should not be disregarded.
